# Draft Genome Sequence of *Bacillus subtilis* Type Strain B7-S, Which Converts Ferulic Acid to Vanillin

**DOI:** 10.1128/genomeA.00025-14

**Published:** 2014-02-13

**Authors:** Peng Chen, Suyue Li, Lei Yan, Ningbo Wang, Xiaojuan Yan, Hongyu Li

**Affiliations:** a Gansu Institute of Business and Technology (GIBT), Lanzhou, People’s Republic of China; b School of Life Sciences, Lanzhou University, Lanzhou, People’s Republic of China; c College of Life Science and Technology, Heilongjiang Bayi Agricultural University, Daqing, People’s Republic of China

## Abstract

The *Bacillus subtilis* type strain B7-S was obtained through induction with ferulic acid. Here, we present the draft genome of strain B7-S, which contains 5,313,924 bp, with a G+C content of 35.8%, 5,135 protein-coding genes, and 40 tRNA-encoding genes.

## GENOME ANNOUNCEMENT

*Bacillus subtilis* is an aerobic, endospore-forming, model organism of Gram-positive bacteria and has been granted generally recognized as safe (GRAS) status (1–3). Here, we report the genome of the *B. subtilis* B7-S strain (CCTCC M 2011162), which can biotransform ferulic acid into vanillin.

The whole-genome sequence of B7-S was obtained using the Illumina HiSeq 2000 sequencing technology by Shanghai Majorbio Bio-pharm Technology Co., Ltd. (Shanghai, China). A library containing 300-bp inserts was constructed. Together, 5,839,802 paired reads, 145,951 single reads, and a total of 1,174,793,050 bases with an average coverage of 221.1× were obtained. The reads were filtered to remove adapter sequences, low-quality bases (Phred score <20), 5′ ends that contain bases that are not A, G, C, or T before shearing, and reads with 10% N to yield adapter and small fragments, with a length of <25 bp after qualitative pruning. The reads were assembled into 72 contigs (contig N_50_, 194,366 bp; contig N_90_, 53,264 bp) and 82 scaffolds (scaffold N_50_, 198,717 bp; scaffold N_90_, 55,898 bp) using the Short Oligonucleotides Alignment Program (SOAP) *de novo* alignment tool (http://soap.genomics.org.cn/). The Glimmer 3.0 software (http://www.cbcb.umd.edu/software/glimmer/) was then used for gene prediction.

The draft genome of B7-S consists of 72 contigs of 5,317,475 bp and has an average G+C content of 35.06%. *B. subtilis* B7-S includes 5,135 protein-coding genes and 40 tRNA-coding genes.

To date, 12 strains of *B. subtilis* genomes have been sequenced. The first published genome sequence of *B. subtilis* was that of strain 168, which contains 4,214,810 bp, with a G+C content of 43.0% and 4,100 protein-coding genes (4, 5). The genome size of B7-S is larger and the G+C content is smaller than that of strain 168. Vanillin can result from the biotransformation of some materials, such as guaiacol, isoeugenol, and ferulic acid (6–9). *B. subtilis* B7-S is capable of biotransforming ferulic acid into vanillin. We previously showed that this strain can tolerate a concentration of ferulic acid of 2.0 g/liter and exhibits a conversion efficiency of 55% to ~63%. Enoyl-coenzyme A (CoA)-hydratase-aldolase (*ech*) is a very important enzyme in the biotransformation of ferulic acid into vanillin; however, vanillate dehydrogenase (*vdh*) can convert vanillin into vanillic acid, and its presence is thus not conducive to the accumulation of vanillin (10, 11). The analysis of the B7-S genome revealed the presence of the *ech* gene and the absence of the *vdh* gene, which may explain the ability of the strain to biotransform ferulic acid into vanillin.

The publication of the genome sequence of *B. subtilis* B7-S, which can convert ferulic acid into vanillin, is of great importance for both basic and applied studies. Further analysis of the genome sequence through genetic engineering techniques may improve the conversion efficiency and the pathway responsible for the biotransformation of ferulic acid into vanillin by the B7-S strain.

### Nucleotide sequence accession numbers.

The sequence of *B. subtilis* B7-S under this whole-genome shotgun project has been deposited at DDBJ/EMBL/GenBank under the accession no. AZNI00000000. The version described in this paper is version AZNI01000000.
